# Supplementary material for: Content-rich biological network constructed by mining PubMed abstracts
Source: BMC Bioinformatics. 2004 Oct 8;5:147. doi: 10.1186/1471-2105-5-147 (PMC528731; doi:10.1186/1471-2105-5-147)
Supplement: Additional File 2 — The original results of the above study (non-essential files are deleted to keep the file size under the limit set by BMC bioinformatics). [file 1471-2105-5-147-S2.bz2 › chilibotAdditionalFile2/dip05/233ID10903799E757/html/IL2RB_IL2RG.html]

 


 **IL2RB** and **IL2RG** 
  
Found 102 abstracts in PubMed, retrieved 05.  
 

 What does Google say? 
 PDF only 
| .edu only 

---

**Interactive relationship** (e.g. stimulation, inhibition, etc)

**Non-interactive relationship** (e.g. studied together, co-existance, homology, etc.)

- Cell surface expression of CD25, CD122  [ **IL2RB** ] , CD132  [ **IL2RG** ] , and CD69 were also determined and no statistical differences were found between both groups.  Ref: 12635575 Allergy Asthma Proc< MedlineTA>Allergy Asthma Proc,
- Cells stained with Annexin V Anx and antibodies to CD56, CD3, CD95, CD25, CD122  [ **IL2RB** ]  or CD132  [ **IL2RG** ]  were examined by flow cytometry.  Ref: 12594840 Eur J Immunol, 2003
- Supporting this view were the substantially diminished levels of IL 2, CD25, CD122  [ **IL2RB** ] , and CD132  [ **IL2RG** ]  mRNA found in AGC10 containing cultures of PHA activated lymphocytes.  Ref: 12467977 Mol Biochem Parasitol< MedlineTA>Mol Biochem Parasitol, 2003
- AGC10 also decreased the intracellular levels of CD25, CD122  [ **IL2RB** ] , and CD132  [ **IL2RG** ]  in CD4 and CD8 cells stimulated with the T cell mitogen phytohemagglutinin PHA.  Ref: 12467977 Mol Biochem Parasitol< MedlineTA>Mol Biochem Parasitol, 2003
- The receptors for interleukin 2 IL 2 and interleukin 15 IL 15 in T cells share the IL 2R beta subunit CD122  [ **IL2RB** ]  and gamma C  [ **IL2RG** ]  subunit but have private alpha subunits.  Ref: 12121224 Immunol Cell Biol, 2002
- Like living Trypanosoma cruzi, its AGC10 membrane glycoprotein inhibits interleukin 2 IL 2 secretion and membrane expression of CD25, CD122  [ **IL2RB** ] , and CD132  [ **IL2RG** ]  the components of the high affinity IL 2 receptor  [ **IL2RB** ]  by activated human lymphocytes.  Ref: 12467977 Mol Biochem Parasitol< MedlineTA>Mol Biochem Parasitol, 2002
